# Prescreening based on the presence of CT-scan abnormalities and biomarkers (KL-6 and SP-D) may reduce severe radiation pneumonitis after stereotactic radiotherapy

**DOI:** 10.1186/1748-717X-5-32

**Published:** 2010-05-09

**Authors:** Hideomi Yamashita, Shino Kobayashi-Shibata, Atsuro Terahara, Kae Okuma, Akihiro Haga, Reiko Wakui, Kuni Ohtomo, Keiichi Nakagawa

**Affiliations:** 1 Department of Radiology, University of Tokyo Hospital, Hongo, Bunkyo-ku, Tokyo, Japan

## Abstract

**Purpose:**

To determine the risk factors of severe radiation pneumonitis (RP) after stereotactic body radiation therapy (SBRT) for primary or secondary lung tumors.

**Materials and methods:**

From January 2003 to March 2009, SBRT was performed on 117 patients (32 patients before 2005 and 85 patients after 2006) with lung tumors (primary = 74 patients and metastatic/recurrent = 43 patients) in our institution. In the current study, the results on cases with severe RP (grades 4-5) were evaluated. Serum Krebs von den Lungen-6 (KL-6) and serum Surfactant protein-D (SP-D) were used to predict the incidence of RP. A shadow of interstitial pneumonitis (IP) on the CT image before performing SBRT was also used as an indicator for RP. Since 2006, patients have been prescreened for biological markers (KL-6 & SP-D) as well as checking for an IP-shadow in CT.

**Results:**

Grades 4-5 RP was observed in nine patients (7.7%) after SBRT and seven of these cases (6.0%) were grade 5 in our institution. A correlation was found between the incidence of RP and higher serum KL-6 & SP-D levels. IP-shadow in patient's CT was also found to correlate well with the severe RP. Severe RP was reduced from 18.8% before 2005 to 3.5% after 2006 (*p *= 0.042). There was no correlation between the dose volume histogram parameters and these severe RP patients.

**Conclusion:**

Patients presenting with an IP shadow in the CT and a high value of the serum KL-6 & SP-D before SBRT treatment developed severe radiation pneumonitis at a high rate. The reduction of RP incidence in patients treated after 2006 may have been attributed to prescreening of the patients. Therefore, pre-screening before SBRT for an IP shadow in CT and serum KL-6 & SP-D is recommended in the management and treatment of patients with primary or secondary lung tumors.

## Introduction

Stereotactic body radiation therapy (SBRT) has been widely used as a safe and effective treatment method for primary or metastatic lung tumors [1]. According to the protocol of Japan Clinical Oncology Group (JCOG) 0403 study [2,3], the absolute contraindication to SBRT was pregnancy. Relative contraindications consisted of (a) a history of irradiation to the concerned site, (b) severe interstitial pneumonitis or pulmonary fibrosis, (c) severe diabetes or connective tissue disease, and (d) common use of steroids. However, these complications preclude other treatment methods in some cases and radiation therapy becomes the only available treatment. Favorable initial clinical results, and local control rates around 90% have been reported [4-10].

Although the mechanisms are not completely understood, it is critical to review the biologic factors involved in radiation lung damage. Current evidence suggests that many factors and various lung parenchymal cells contribute to the pathogenesis of radiation lung damage [11]. The progression of radiation-induced damage is the result of an early activation of an inflammatory reaction leading to the expression and maintenance of an elevated cytokine cascade [12]. Kong *et al*. [13] concluded that blood biomarkers such as transforming growth factor (TGF)-beta1, interleukin (IL)-6, krebs von den Lungen-6 (KL-6), surfactant proteins (SP), and IL-1ra could accurately predict radiation-induced lung damage. Serum KL-6 and SP-D were also evaluated as predictive biomarlers for radiation pneumonitis (RP) in this study.

For normal tissues, the use of a single dose rather than a conventional fractionated dose can increase the risk of complications. However, few cases with severe toxicity have been reported [14-16]. In the current study, cases of severe RP (grades 4-5) that received SBRT for lung tumors in our institution were evaluated. In our previous report [17], the overall incidence rate of grades 2-5 RP was 29% (7/25 cases) and three patients (12%) died from RP from May 2004 to April 2006 at the median follow-up time of 18 months after completing SBRT. A significant decrease of the incidence rate of severe RP was observed for the period entering into 2006. The purpose of this study was to determine the risk factors of severe RP after SBRT for primary or secondary lung tumors.

## Methods and materials

### Subjects

From January 2003 to March 2009, SBRT was performed on 117 patients with lung tumors in our institution. SBRT was performed for primary lung cancers in 74 cases (63%) and for metastatic or recurrent lung tumors in 43 cases (37%) (Table 1). These consecutive 117 patients were evaluated retrospectively. There were 98 males and 19 females, and the median age was 72 years (range; 28-84 years). Thirteen patients (11%) had a shadow of interstitial pneumonitis (IP) in the CT before SBRT, 23 patients (20%) had high serum KL-6 value, and 19 patients (16%) had high SP-D value. The upper limit of serum KL-6 and SP-D was defined as 500.0 U/mL and 110.0 ng/mL, respectively.

**Table 1:** Characteristics of the tumor

Subject	N	(%)
Biopsy proved primary lung cancer	60	51
cT1N0M0	39	33
cT2N0M0	19	16
the others	2	2
Unconfirmed histology (suspected of primary lung cancer)	14	12
Metastatic or recurrent lung cancer	43	37
		
Total	117	100

All patients enrolled in this study satisfied the following eligibility criteria: a) solitary or double lung tumors; b) tumor diameter < 40 mm; c) no evidence of regional lymph node metastasis; d) Karnofsky performance status scale > or = 80%; and e) tumor not located adjacent to major bronchus, esophagus, spinal cord, or great vessels. Patients with an active malignant lesion other than lung were excluded. Therefore, no chemotherapy was combined with SBRT. There were 32 patients (27%) who were treated before 2005. After 2006, patients with a high risk for RP who had an obvious IP shadow on CT with a 3-mm slice before SBRT together with a high value of serum KL-6 & SP-D were excluded from receiving SBRT.

In the high resolution chest CT, IP shadow was defined as a mandatory observation beneath the pleura and a honeycomb lung. IP shadows were graded by their radiographically estimated total lung volume as follows: slight, less than 10%; moderate, 10-50%; and severe, >50%.

### Planning procedure and treatment

**Figure 1:**
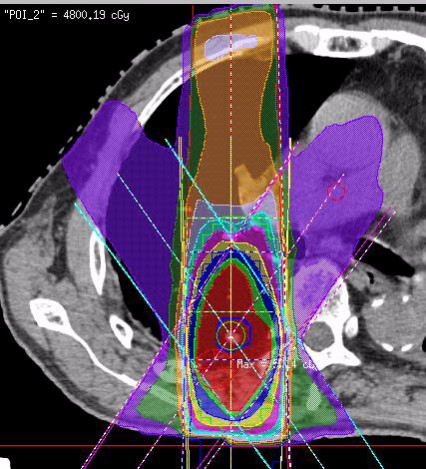
**An example of dose distribution of SBRT (Pt. No. 5)**.

**Figure 2:**
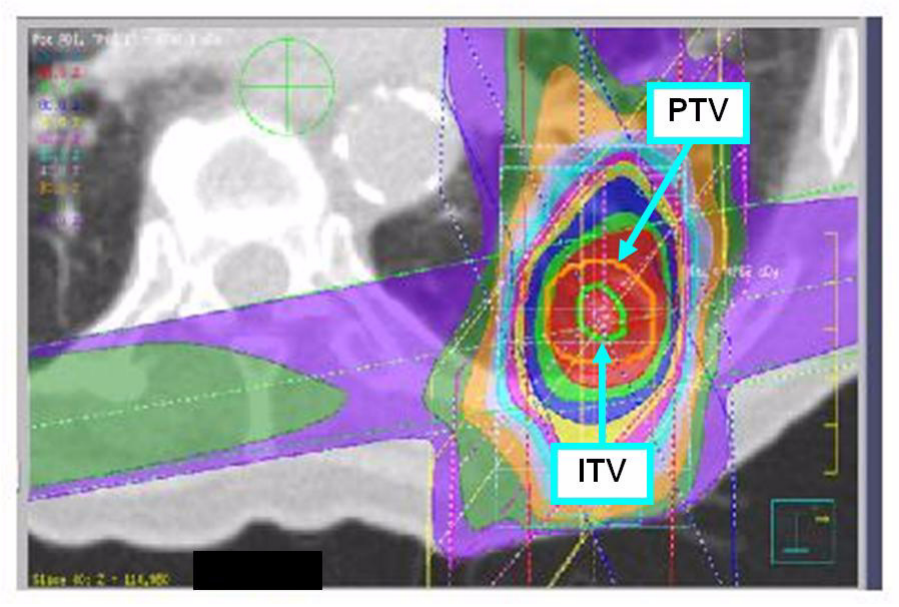
**An example of dose distribution of SBRT (Pt. No. 7)**.

**Figure 3:**
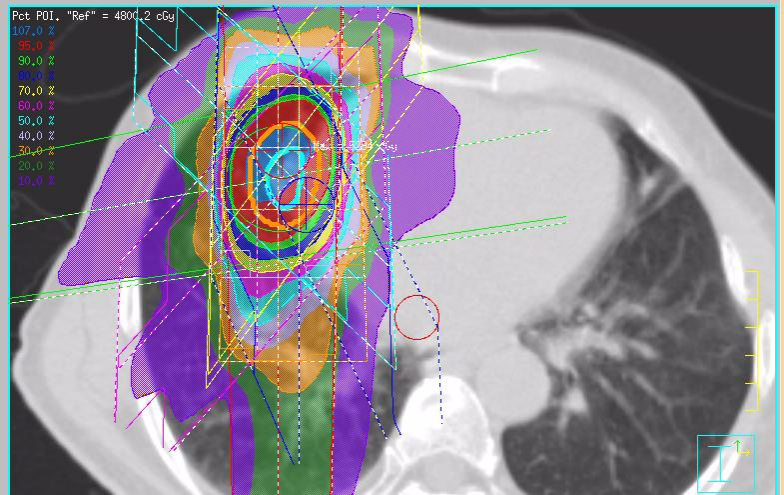
**An example of dose distribution of SBRT (Pt. No. 8)**.

The treatment methods which included the definition of the internal target volume (ITV) were performed according to JCOG 0403 phase II protocol [2,3]. The following gives a brief description of the treatment methods, which were described in detail in our previous report [17]. SBRT was performed daily with a central dose of 48 Gy in four fractions over 4-8 days. Each CT slice was scanned with an acquisition time of four seconds to include the whole phase of one respiratory cycle. The axial CT images were transferred to a 3-dimension RT treatment-planning machine (Pinnacle3, New Version 7.4i, Philips). Spicula formation and pleural indentation were included within the ITV. The mediastinal lymph nodes were not included from the irradiation field. The setup margin (SM) between ITV and the planning target volume (PTV) was 5 mm in all directions. There was an additional 5 mm leaf margin to PTV, according to JCOG0403 protocol, in order to make the dose distribution within the PTV more homogeneous. No pairs of parallel opposing fields were used. The target reference point dose was defined at the isocenter of the beam. The iso-dose distribution of an SBRT treatment was shown in Figures 1,2,3.

The dose limitation for pulmonary parenchyma was mean lung dose (MLD) < 18.0 Gy, percentage of total lung volume receiving greater than or equal to 20 Gy (V20) < 20%, and V15 < 25% according to JCOG0403 protocol.

### Radiation method

**Figure 4:**
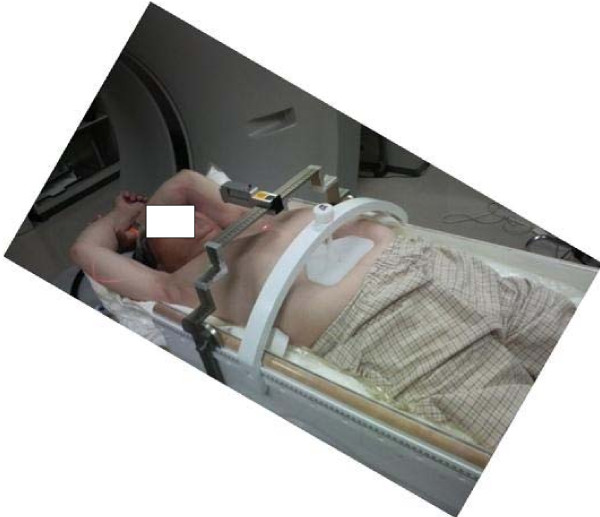
**Body frame and abdominal pressure board**.

SBRT was given in at least 8 ports by linear accelerator (Elekta Synergy System, Elekta Ltd, Crawley, UK) after the Synergy system was available in our institution from February 2007. At least eight beams (I-rotation angle was 0 degree only in two beams) were used. CT verification of the target isocenter was performed before each treatment session using a kilovoltage-based cone-beam CT (CBCT) unit in the same room and in a treatment position. The Linac machine was Elekta Synergy with the cone-beam CT. The details of the radiation method before 2007 were described in our previous report [17]. The collapsed cone (CC) convolution method in Pinnacle^3 ^was used as the heterogeneous correction method for lung. The breathing suppression was done with a body frame and an abdominal pressure board (Figure 4).

### Definition of RP grading

The toxicity data were collected retrospectively from the patient files. Basically, the RP grading system used followed the Common Terminology Criteria for Adverse Events (CTCAE) v3.0, and the grades were as follows: Grade 1, asymptomatic (radiographic findings only); Grade 2, radiographic findings plus symptomatic and not interfering with activities of daily living (ADL); Grade 3, radiographic findings plus symptomatic and interfering with ADL or O2 indicated; Grade 4, radiographic findings plus life-threatening (ventilatory support indicated), and Grade 5, radiographic findings plus death. Patients with mild pulmonary CT changes after SBRT were categorized as Grade 1. The radiographic findings common to the 5 grades were (a) shadow distribution just beneath pleura, (b) honeycomb lung, (c) traction bronchitis/dilation of small bronchus, (d) ground-glass opacity (GGO), or (e) infiltrative shadow (consolidation), which was not recognized in the CT before SBRT.

### Follow-up

CT exams with 3-mm slices were performed at 2, 4, 6, 9, 12, 15, 18, and 24 months after SBRT for asymptomatic patients. Additionally, on the same day as CT, serum KL-6, SP-D, white blood cell (WBC), lactate dehydrogenase (LDH), C-reactive protein (CRP), and tumor markers were measured in the blood plus an oxygen saturation was measured from a fingertip.

### Statistical Analysis

The relationship between G4-5 RP and pre-SBRT factors was compared with the X^2 ^test. The cumulative probability of RP was calculated and drawn applying the Kaplan-Meier algorithms with day of treatment as the starting point. Subgroups were compared using log-rank statistics. Values of p < 0.05 were considered statistically significant. Statistical calculations were conducted using version 5.0 StatView software (SAS Institute, Cary, NC).

## Results

The median follow up time for all 117 patients was 14.7 months (range; 0.3-76.2 months). The control rate within the radiation field was 86.3% (101/117 cases).

**Figure 5:**
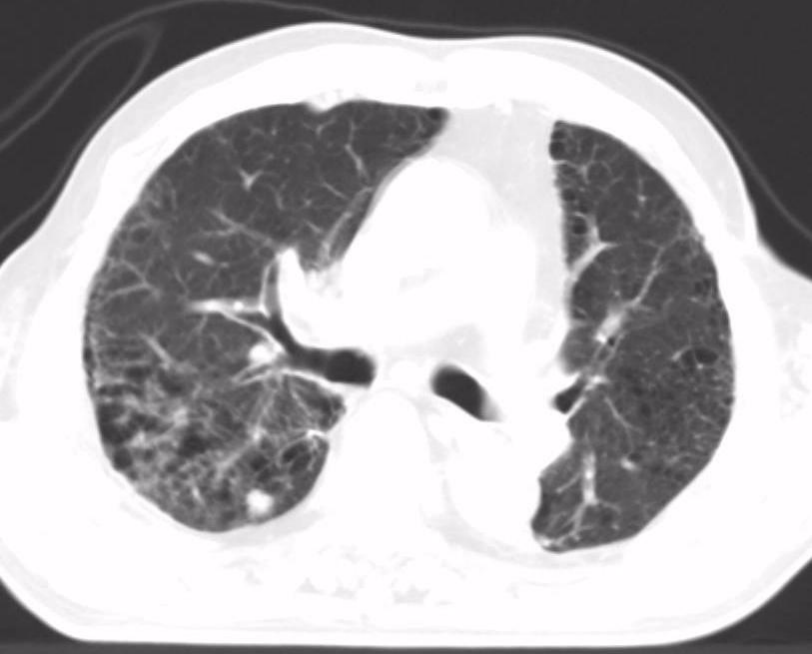
**CT images before SBRT (Pt. No. 5)**.

**Figure 6:**
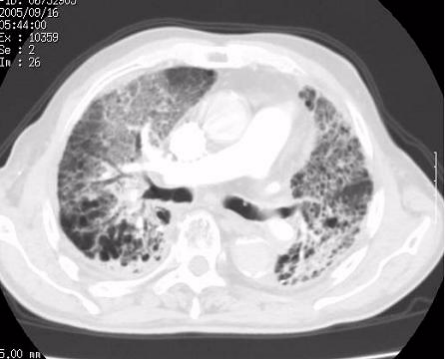
**CT images of radiation pneumonitis after SBRT (Pt. No. 5)**. The finding was acute exacerbation of IP.

**Figure 7:**
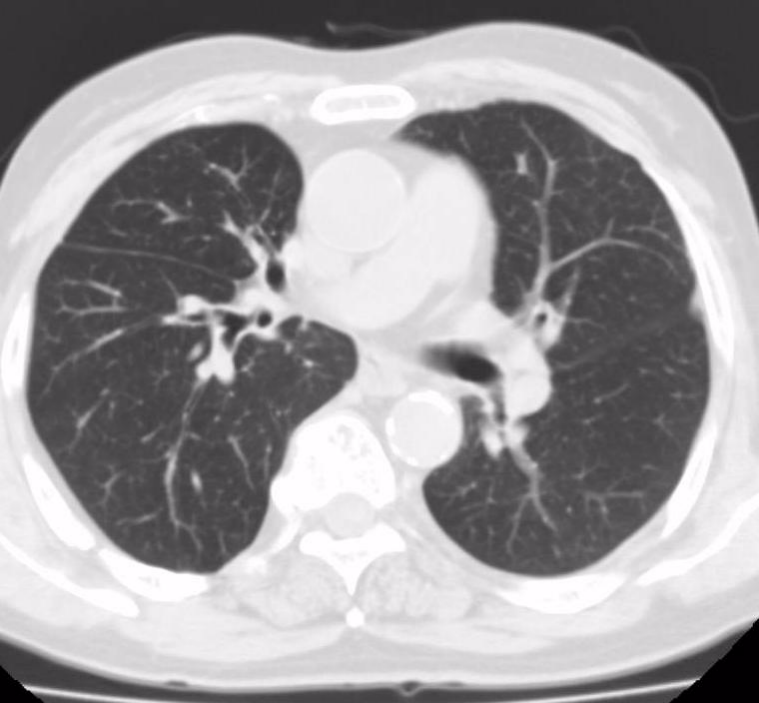
**CT images before SBRT (Pt. No. 7)**.

**Figure 8:**
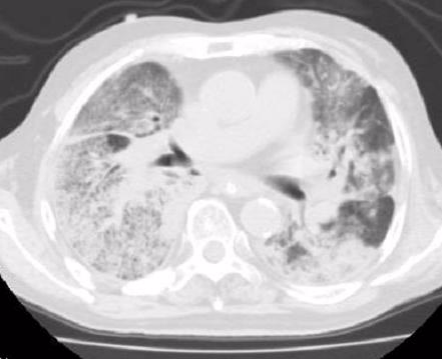
**CT images of radiation pneumonitis (acute exacerbation of IP) after SBRT (Pt. No. 7)**.

**Figure 9:**
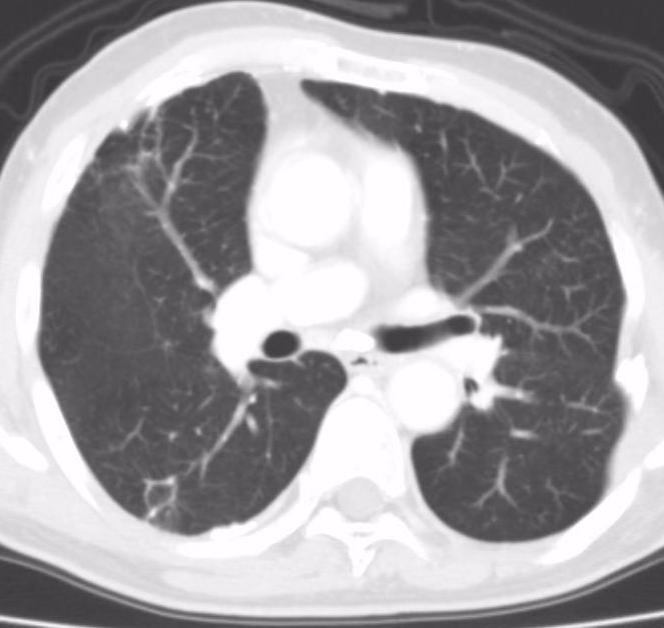
**CT images before SBRT (Pt. No. 8)**.

**Figure 10:**
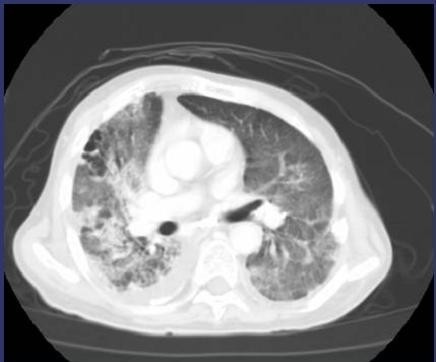
**CT images of radiation pneumonitis (acute exacerbation of IP) after SBRT (Pt. No. 8)**.

**Figure 11:**
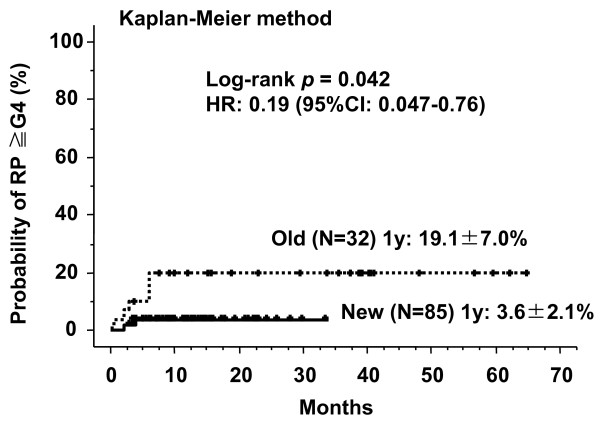
**Cumulative probability curves of severe radiation pneumonitis of grades 4-5 divided by pre-2005 (old group) and post-2006 (new group)**.

RP of grade 4 or higher was observed in nine patients (7.7%) and the median time of showing symptoms was 4.0 months (range; 0.4-6.0 months) (Table 2). All of these nine RPs were due to acute exacerbation of IP (Figures 5,6,7,8,9,10) and steroid pulse therapy combined with an oral anti-pneumocystis carinii drug was administered to these patients. Grade 4 RP with intubation was seen in two cases and the other seven cases were grade 5. Grade 3 RP was seen in two patients during this time period. Grade 4 or higher RP was noted in six out of 32 patients (18.8%) before 2005 and in only three out of 85 patients (3.5%) after 2006 (Figure 11). This difference had a statistical significance (log-rank *p *= 0.042 and X^2 ^*p *= 0.018).

**Table 2:** Characteristics of nine patients with G4-5 of RP

**Case No**.	s KL-6	S SP-D	IP shadow	RP grading	Onset time	State	V20 (%)	V40 (%)	MLD (cGy)	Stage	PTV (cc)	D95 (Gy)	Location	
1	950↑	286↑	moderate	5	3.0 Mo	Postoperative	6.7	2.7	938	IV	26.4	46.29	Lt	hilum
2	582↑	95	slight	5	2.0 Mo	Fresh	7.6	1.9	568	IA	47.5	45.57	Lt	hilum
3	852↑	136↑	severe	5	6.0 Mo	Postoperative	11.2	4.6	791	IV	120.9	45.00	Rt	S6
4	1590↑	NA	slight	5	6.0 Mo	Fresh	5.6	1.9	426	IA	29.4	44.05	Rt	S10
5	NA	NA	(-)	4	0.4 Mo	Fresh	5.0	1.5	291	IA	42.5	47.80	Lt	S8
6	289	101	slight	5	5.9 Mo	Fresh	7.0	2.0	440	IA	56.5	48.90	Rt	S10
7	497	321↑	(-)	4	4.0 Mo	Postoperative	2.6	0.9	269	IV	7.7	45.48	Lt	S10
8	833↑	135↑	slight	5	2.1 Mo	Fresh	6.3	2.3	492	IA	20.9	47.62	Rt	S5
9	883↑	235↑	slight	5	1.0 Mo	Fresh	3.7	0.7	288	IB	23.9	44.80	Rt	S2
	(0-500)	(0-110)												

*Abbreviation *; NA = not available

Serum KL-6 was determined in 8 of the 9 patients with grades 4-5 RP and in 95 of the 108 patients with grades 0-3 RP. Of the 8 patients with grades 4-5 RP, serum KL-6 (U/mL) was elevated in 6 patients (75%) (Table 2). Serum SP-D was determined in 7 patients with grades 4-5 RP and in 93 patients with grades 0-3 RP. Of the 7 patients with grades 4-5 RP, serum SP-D (ng/mL) was evaluated in 5 patients (71%) (Table 2). Additionally, the IP shadow was seen in seven cases (78%) in the CT before SBRT within or outside of radiation field. The radiation dose prescribed was within the protocol in all 117 patients. The appearance of grades 4-5 RP and serum KL-6 value (1-year cumulative incidence; 32% vs. 3% and log-rank *p *< 0.0001 & X^2 ^*p *= 0.0002), SP-D value (1-year; 29% vs. 3% and log-rank *p *= 0.0001 & X^2 ^*p *= 0.0002), or IP shadow in CT before SBRT (1-year; 57% vs. 2% and log-rank *p *< 0.0001 & X^2 ^*p *< 0.0001) showed positive correlations (Table 3).

**Table 3:** Relationship between G4-5 RP and pre-SBRT factors

Pre-SBRT factors	G4-5 RP	G0-3 RP	Total	X2 test	1-year cumulative incidence of G4-5 RP	log-rank
Serum KL-6						
high value	6	17	23	*p *= 0.0002	32%	*p *< 0.0001
within normal level	2	78	80		3%	
not available	1	13	14			
Serum SP-D						
high value	5	14	19	*p *= 0.0002	29%	*p *= 0.0001
within normal level	2	79	81		3%	
not available	2	15	17			
IP shadow in CT						
(+)	7	6	13	*p *< 0.0001	57%	*p *< 0.0001
(-)	2	102	104		2%	

The risk factors of RP other than serum KL-6, SP-D, and IP shadow in CT are shown in Table 4. The mean PTV for nine patients with severe RP was 29.4 cc (range: 7.7-120.9 cc) and was 42.5 cc (range: 7.5-239.4 cc) of for the other low-grade RP patients. None of these risk factors were different for those patients with and without grades 4-5 RP.

**Table 4:** Risk factors of severe RP

	Patients with G4-5 RP	Patients without G4-5 RP	*p *value
Total	9 (8%)	108 (92%)	
Patient specific factors			
Pulmonary function			
VC (L)			
mean +/- SD	3.27 +/- 0.65	2.75 +/- 0.85	*N.S*.
range	2.76-4.01	1.54-4.07	
FEV 1.0 (L)			
mean +/- SD	2.11 +/- 0.68	1.87 +/- 0.82	*N.S*.
range	1.59-3.24	0.59-3.24	
K-PS (%)			
90	5 (56%)	52 (48%)	*N.S*.
80	4 (44%)	56 (52%)	
Age (y)			
mean +/- SD	73.3 +/- 6.8	70.1 +/- 14.1	*N.S*.
range	68-80	24-93	
COPD			
With	2 (22%)	22 (20%)	*N.S*.
Without	8 (78%)	86 (80%)	
Treatment specific factors			
Size of the PTV (cc)			
mean +/- SD	29.4 +/- 33.2	42.5 +/- 13.7	*N.S*.
range	7.7-120.9	7.5-239.4	
Mean lung dose (Gy)			
mean +/- SD	5.0 +/- 2.3	4.2 +/- 1.4	*N.S*.
range	2.7-9.4	1.7-7.9	
Lung V20 (%)			
mean +/- SD	5.9 +/- 2.7	5.8 +/- 2.6	*N.S*.
range	2.6-11.2	1.0-11.0	
Target location			
Central	2 (22%)	17 (16%)	*N.S*.
Peripheral	7 (78%)	91 (84%)	

*Abbreviation*:

COPD = chronic obstructive pulmonary diseases

RP = radiation pneumonitis

G4-5 = grades 4-5

PTV = planning target vlume

FEV = Forced expiratory volume

K-PS = Karnofsky Performance status

N.S. = not significant

## Discussion

This was a retrospective study to evaluate the incidence rate and risk factors of severe RP after SBRT for primary (74 patients), metastatic and recurrent (43 patients) lung tumors. Grades 4-5 RP were noted in 9 patients (7.7%); IP shadow in the CT, and high serum KL-6 & SP-D values before SBRT showed positive correlations with grades 4-5 RP. Seven of the 117cases (6.0%) were of grade 5 in our institution. After 2006, severe grades 4-5 RP were significantly reduced.

According to Borst *et al*. [15], the crude incidence rate of grade 2 RP was 10.9% for the SBRT on 128 patients with malignant pulmonary lesions who were treated with 6-12 Gy per fraction with a median MLD of 6.4 Gy (range: 1.5-26.5 Gy). According to Rusthoven *et al*. [16], grades 2-3 RP was rare, occurring in only one out of 38 patients (2.6%) with one to three lung metastases after SBRT of 48-60 Gy in 3 fractions. They used the dose constraint of V15 < 35%. According to Nagata *et al*. [1], no severe symptomatic pulmonary complications (NCICTC Grade 3 or larger) were encountered. Timmerman *et al*. [14] reported in 2006 that a SBRT treatment dose of 60-66 Gy total in three fractions was administered during 1 to 2 weeks for 70 patients with clinically staged T1-2N0M0 (tumor size < or = 7 cm) biopsy-confirmed non-small cell lung cancer (NSCLC). This resulted in toxicity of grades 3 to 5 in a total of 14 patients (20%) and grade 5 was seen in four patients (5.7%). Le QT *et al*. [18] reported in 2006 that after single-fraction SBRT (15-30 Gy) was performed for 32 patients (21 NSCLC and 11 metastatic tumors), two patients (6%) suffered from RP of grade 5.

Moreover, according to Rusthoven *et al*. [16], patients were required to have adequate lung function, which was defined as stable arterial hemoglobin saturation above 90% with minimal exertion, forced expiratory volume (FEV) of 1.0% higher than the predicted value of 40% or more than 1 L and carbon monoxide diffusing capacity (DLCO) higher than the predicted 40% value. In our institution, the exclusion criteria of SBRT consisted of an FEV of 1.0% at less than 750 mL, and an obvious IP shadow on the roentgen examination according to JCOG 0403 protocol.

RP of grades 4-5 occurred in six out of 32 patients (18.8%) before 2005 and in only three out of 85 patients (3.5%) after 2006 (Figure 11). The significant reduction of severe grades 4-5 RP after 2006 in our institution is believed to be due to the selection of appropriate patients. After 2006, patients were excluded from SBRT if they had an obvious IP shadow on the CT-scan (slice thickness 3.0 mm), and if serum KL-6 and SP-D levels were high. All of the severe RP cases in our institution consisted of acute exacerbation of IP outspreading over the radiation field. Admittedly, these nine patients with severe RP represent a small sample. Whether our results are a coincidence that biomarkers and CT shadows are indeed significantly different in patients with grades 4-5 toxicity compared to patients without RP awaits confirmation in further studies.

KL-6 is the indicator that specificity is high for IP and is clinically evaluated for the purpose of diagnosing IP. In addition, KL-6 is important as an index of the activity of IP because it becomes significantly high for IP with activity. In the human body, KL-6 does not develop in a type I alveolus epithelial cell. However, KL-6 develops in a type II alveolus epithelial cell, in a bronchial epithelial cell, and in a bronchus gland cell. The expression of KL-6 increases in the hyperplasia of the type II of alveolus epithelial cell in IP. A small quantity of KL-6 is present in the liquid coating the alveolus in normal lungs, and its density increases during hyperplasia of the type II alveolus epithelial cell for IP. In addition, because inflammation occurs, blood vessel permeability rises, and KL-6 in the alveolus coating liquid shifts easily into the blood. As a result, KL-6 in the blood rises in the IP. When an injury to the lung stroma is evaluated, KL-6, SP-A, SP-D, and MCP-1 are examined. Of these, there is a report that KL-6 was highest in both sensitivity (93.9%) and specificity (96.3%) [19]. Furthermore, SP-D levels at 50 to 60 Gy (midway during radiation therapy) showed greater sensitivity and positive predictive values for RP detection (74% and 68%, respectively) than SP-A (26% and 21%, respectively) [20].

## Conclusion

The frequency of severe RP in our institution has recently shown a decrease, by prescreening patients for serum KL-6 and SP-D as biomarkers of severe RP. When SBRT was performed on patients presenting with an IP shadow in CT and a high value of serum KL-6 before treatment, severe radiation pneumonitis occurred at a high rate. Therefore, pre-screening of patients before SBRT appears to be a useful strategy in treating lung tumors.

## Authors' contributions

HY collected and analyzed data and performed statistical analysis. HY and SK-S drafted the manuscript. AT, KO, AH, and RW reviewed the data and revised the manuscript. KO and KN designed the study and revised the final version. All authors have read and approved the final version of the manuscript.

## Competing interests

The authors declare that they have no competing interests.
